# A Natural History of Infective Endocarditis, Preceded by Decompensated Chronic Liver Disease and Severe Community-Acquired Pneumonia: Indictment in Four Parts and with Right of Reply

**DOI:** 10.3201/eid1810.AD1810

**Published:** 2012-10

**Authors:** Nancy L. Merridew

**Affiliations:** The Alfred Hospital, Carlton, Victoria, Australia

**Keywords:** endocarditis, bacteria, liver cirrhosis, alcoholic, hepatitis, viruses, human, respiratory tract infections, pneumonia, humanities

## *ONE.* *Trespass*

His stalwart skin, sallow armor,

harbored memories and marks

of a difficult life.

His grave affliction, already perilous,

he endured alone—

ostracized by family and society.

His swollen belly, crazed by Medusa,

pouted taut as artificial breaths

stretched rankled lungs.

His seasoned vessels, illicit euphoria’s labyrinth,

adorned with plastic portals

infused antidote.

His cloying blood, claret lately poisoned,

delivered assassin to lair—

Duke in silent carriage alighted at the chamber.

** ** ** ** **

## *TWO.* *Malice Aforethought*

His haughty foe, ignoble despite the Criteria,

stole a vantage of supreme command

at the Greatest Vessel door.

His insistent heartbeat, two sounds once discreet,

in rhythmic phrases murmured

remarks of furtive progress.

His ardent doctor, genius clinician,

lifted stethoscope to precordium—

message received.

His pliant pulses, company accommodated—

Watson struck and shuddered,

Corrigan thumped and ebbed.

His teeming veins, mercurial blood extracted,

yielded Duke’s identity

for precise retaliation.

His shriveled Liver, nominally ironic,

sacrificed to intoxicated sanctuary—

Child-Pugh C proscribed surgical reprieve.

His adept assailant, though exposed for battle,

abetted by fickle liver

retained advantage for the coup.

** ** ** ** **

## *THREE.* *Progressive Assault*

His heart, excavated by festering mercenaries,

inexorably leached

their venomous arsenal.

His body, conscripted slave to advancing legion,

appointed satellite outposts—

Streptococcal Empire rising.

His scent, previously acrid,

drifted metallic—

life corroding.

His brain, shadowed by grim delirium;

encephalopathic, embolic, prophetic—

entombed within cranial vault.

His eyelids, fluttering shrouds,

veiled infinite irises, icteric globes and

retinas maligned by immunity.

His urine, once flaxen,

oozed vermillion—

tributary of beckoning Rubicon.

His skin, chameleon canvas—

flushed lesions and jaundice

defaced faded tattoos.

His fingers, pulps mottled carmine,

uniquely etched tips

reached to probe oblivion.

His nail-beds, fleshy Rosetta,

signaled the secret

in flecked hieroglyphs.

His palms, unaccustomed to prayer,

denied redemption—

pocked, they too bore stigmata.

** ** ** ** **

## *FOUR.* *Homicidal Ascendancy*

His surgical saviors, glinting mirage,

could offer no cure

except hastened demise.

His thwarted physicians, ashen surrender—

our elixirs no match

for the adversary.

His recalcitrant Duke, relentless,

accomplished unsavory

feats of cardiac passage.

His core engine, chambers thus connected,

ramped to fuel abscessed conduit—

blood roared relentlessly.

His slumped neck, kinetically possessed—

terminally mutilated muscle’s

futile beats rocketed and ricocheted.

His uncanny figure, cadaveric,

inevitably bloated;

Starling forces prevailed.

His sodden lungs, emancipated from machines,

hissed and spluttered

beneath swelling king tide.

His dying breaths, labored then whispered,

dim light obscured final chest fall—

curtains drawn, despite the summer sun.

His only visitor, a community case worker,

absent that day.

Alone when he succumbed to the darkness.

** ** ** ** **

## Audi Alteram Partem

Virulent villain—do you admire your conquest?

